# Leader peptide or pro-segment mutants of renin are misrouted to mitochondria in autosomal dominant tubulointerstitial kidney disease

**DOI:** 10.1242/dmm.049963

**Published:** 2023-06-07

**Authors:** Céline Schaeffer, Maurizio De Fusco, Elena Pasqualetto, Caterina Scolari, Claudia Izzi, Francesco Scolari, Luca Rampoldi

**Affiliations:** ^1^ IRCCS Ospedale San Raffaele, 20132 Milan, Italy; ^2^ Vita-Salute San Raffaele University, 20132 Milan, Italy; ^3^ Division of Nephrology and Dialysis, Department of Medical and Surgical Specialties, Radiological Sciences, and Public Health, University of Brescia and ASST-Spedali Civili of Brescia, 25123 Brescia, Italy; ^4^ Medical Genetics Clinic, Department of Obstetrics and Gynaecology, ASST Spedali Civili, 25123 Brescia, Italy

**Keywords:** Renin, Mitochondria, Trafficking defect, Signal sequence, Autosomal dominant tubulointerstitial kidney disease

## Abstract

Autosomal dominant tubulointerstitial kidney disease (ADTKD), a rare genetic disorder characterised by progressive chronic kidney disease, is caused by mutations in different genes, including *REN*, encoding renin. Renin is a secreted protease composed of three domains: the leader peptide that allows insertion in the endoplasmic reticulum (ER), a pro-segment regulating its activity, and the mature part of the protein. Mutations in mature renin lead to ER retention of the mutant protein and to late-onset disease, whereas mutations in the leader peptide, associated with defective ER translocation, and mutations in the pro-segment, leading to accumulation in the ER-to-Golgi compartment, lead to a more severe, early-onset disease. In this study, we demonstrate a common, unprecedented effect of mutations in the leader peptide and pro-segment as they lead to full or partial mistargeting of the mutated proteins to mitochondria. The mutated pre-pro-sequence of renin is necessary and sufficient to drive mitochondrial rerouting, mitochondrial import defect and fragmentation. Mitochondrial localisation and fragmentation were also observed for wild-type renin when ER translocation was affected. These results expand the spectrum of cellular phenotypes associated with ADTKD-associated *REN* mutations, providing new insight into the molecular pathogenesis of the disease.

## Teaser

**Summary:** Mutations in the renin leader peptide or pro-segment associated with autosomal dominant tubulointerstitial kidney disease lead to misrouting of the mutated protein to mitochondria.

## INTRODUCTION

Renin (encoded by *REN*) is an aspartyl protease present in the blood and responsible for the cleavage of angiotensinogen to produce angiotensin I. It is thus involved in the first step of the renin-angiotensin system, which regulates blood pressure by controlling fluid and electrolyte balance and systemic vascular resistance (Sparks et al., 2014). Renin is synthesised as preprorenin, which is translocated into the endoplasmic reticulum (ER), where it is processed to prorenin, its inactive precursor, by cleavage of the leader peptide. Whereas prorenin is produced in many tissues, mature renin, produced by cleavage of the pro-segment, is exclusively produced in and secreted by renal juxtaglomerular cells (Paul et al., 2006).

Mutations in *REN* (1q32) have been associated with two different diseases. Homozygosity or compound heterozygosity for loss-of-function mutations, most commonly causing complete loss of renin synthesis, are associated with renal tubular dysgenesis (MIM#267430), a rare recessive condition characterised by perinatal mortality (Gribouval et al., 2005). In contrast, dominant mutations are associated with *REN*-related autosomal dominant tubulointerstitial kidney disease (ADTKD-*REN*) (MIM#613092) (Živná et al., 2009), leading to renal tubulointerstitial damage and progressive chronic kidney disease. Dominant *REN* mutations likely have a gain-of-function effect, as in heterozygous carriers of *REN* loss-of-function recessive mutations, the lack of one renin functional allele is not sufficient to induce ADTKD (Gubler and Antignac, 2010). Additional genes have been identified associated with ADTKD, such as uromodulin (*UMOD*), mucin-1 (*MUC1*), *HNF1B* and α1-subunit of translocon 61 (*SEC61A1*), with evidence supporting further genetic heterogeneity (Bleyer et al., 2017; Devuyst et al., 2019). ADTKD is one of the most common monogenic kidney diseases, with an estimated prevalence of ∼3% of patients with end-stage renal disease of genetic origin for ADTKD-*UMOD* alone (Groopman et al., 2019).

ADTKD-*REN* mutations reported to date are non-truncating, missense changes, in-frame deletion affecting residues within the protein leader peptide and missense changes located in the pro-segment or the mature part of the protein (Živná et al., 2020). Interestingly, some relevant differences were reported in the clinical picture of patients bearing mutations in the leader peptide and the pro-segment compared to that of patients with mutations in the mature part of renin. Mutations in the renin leader peptide or pro-segment lead to very early onset of clinical manifestations. Impaired kidney function and hypo-proliferative anaemia with low haemoglobin concentration can indeed be observed as early as 3 or 4 years of age. Anaemia resolves as the child enters adolescence, as long as renal function is not severely impaired. Hyperuricemia and gout usually develop in the second decade of life. Chronic kidney disease is slowly progressive and affected subjects reach end-stage kidney disease in the fifth or sixth decade of life. Mutations in the mature part of renin are associated with a milder renal phenotype, mainly characterised by late onset of kidney disease, hyperuricemia and gout. Indeed, the first signs of renal dysfunction appear in adulthood, at 50-60 years of age, and end-stage kidney disease is reached on average at the age of 70. Infantile hypo-proliferative anaemia was not reported in these patients (Živná et al., 2020).

Studies in cell models showed that all analysed ADTKD-*REN* mutations led to absent/decreased secretion, although their reported effect on intracellular maturation and trafficking was different. Extensive *in vitro* characterisation has been performed for the p.L381P isoform, a mutant of the mature protein, showing complete ER retention and induction of the unfolded protein response (Schaeffer et al., 2019). ER accumulation has also been confirmed for additional mutants in the mature part of renin (Živná et al., 2020). Mutant isoforms in the pro-segment showed partial accumulation in the ER-to-Golgi intermediate compartment (ERGIC) (Živná et al., 2020), whereas mutations in the leader peptide are associated with defective translocation in the ER and intracellular accumulation (Živná et al., 2009, 2020). Despite the apparently different cellular phenotype, mutations in the renin leader peptide and pro-segment are associated with similar disease features, raising the question as to whether they share a yet unravelled cellular phenotype.

In this study, we report a detailed *in vitro* characterisation of mutant renin isoforms in the leader peptide and the pro-segment. We show that these mutations lead to complete or partial mistargeting of renin to the mitochondria, which is accompanied by mitochondrial import defect and fragmentation of the mitochondrial network. This effect is due to the presence of the mutated leader peptide and/or of the pro-segment region; it does not depend on the mature renin protein or activity. These results represent an important additional step towards the current understanding of ADTKD-*REN* pathogenesis and suggest that different primary cellular responses could be the basis of the different ages of disease onset observed in patients.

## RESULTS

### *In silico* prediction of the effect of mutations in the renin leader peptide and pro-segment

**Fig. 1.**
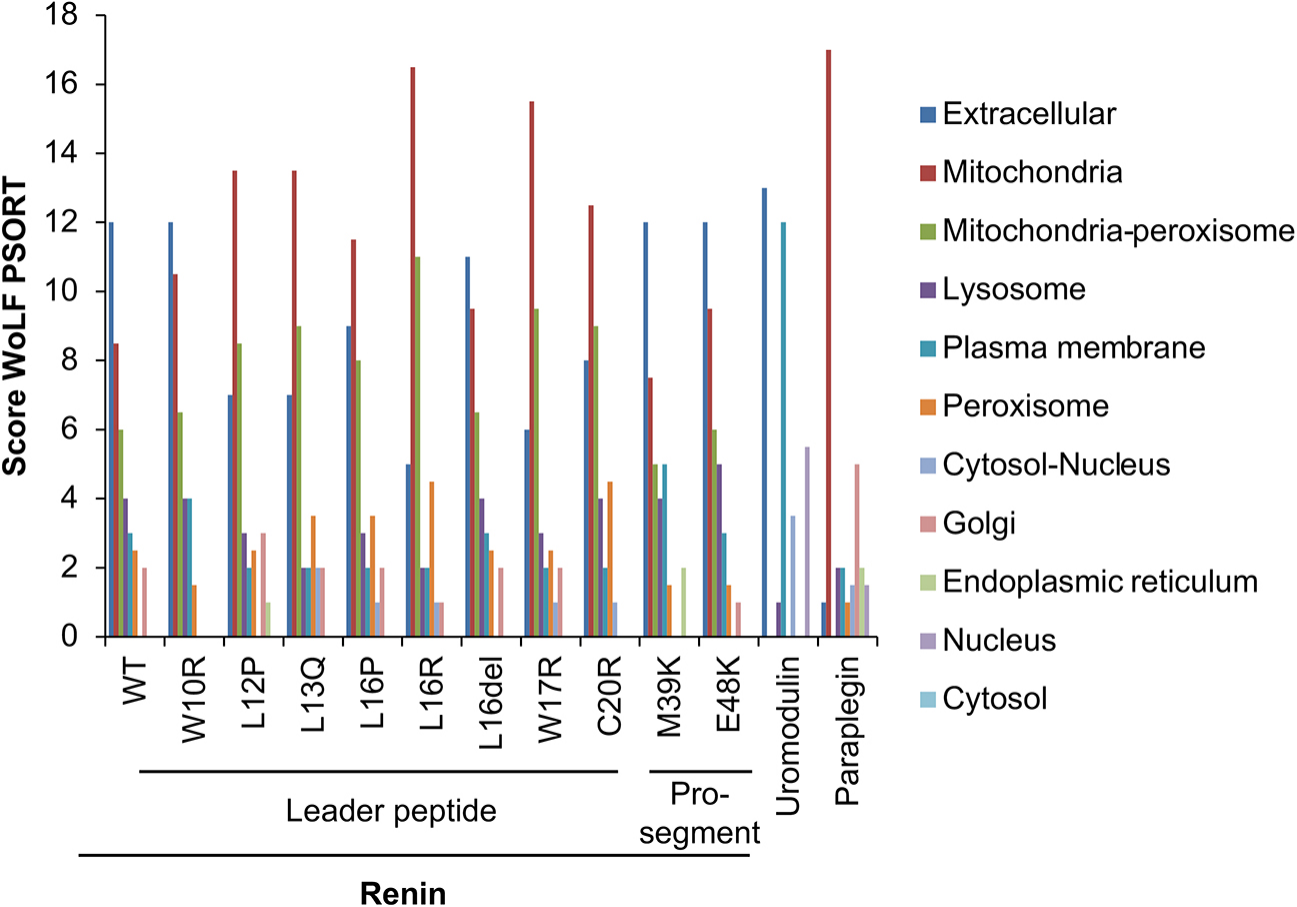
**Prediction of the subcellular localisation of the indicated renin isoforms.** Prediction was performed using the software WoLF PSORT. Uromodulin and paraplegin are shown as examples of a secreted and mitochondrial protein, respectively. Renin mutant isoforms p.L12P, p.L13Q, p.L16P, p.L16R, p.W17R and p.C20R have the highest score for mitochondrial localisation, whereas WT renin is predicted to be secreted. No clear effect is observed for mutations in the leader peptide W10R and L16del or in the pro-segment M39K and E48K, for which the prediction is similar to WT renin.

Stemming on the hypothesis that mutations in the renin leader peptide could interfere with protein localisation, we performed *in silico* analysis using the software WoLF PSORT, which predicts the subcellular localisation of proteins based on their amino acid sequence. Although, as expected, wild-type (WT) renin is predicted to be secreted (extracellular), six out the eight described mutants of the leader peptide are predicted to be localised in the mitochondria (Fig. 1). The predicted localisation of mutants of the pro-segment is similar to that of the WT protein. Uromodulin and paraplegin (encoded by *SPG7*) are shown as examples of secreted and mitochondrial proteins, respectively, the localisation of which is correctly predicted by the software.

### Expression of renin mutant isoforms in different cellular systems unravels misrouting to mitochondria

**Fig. 2.**
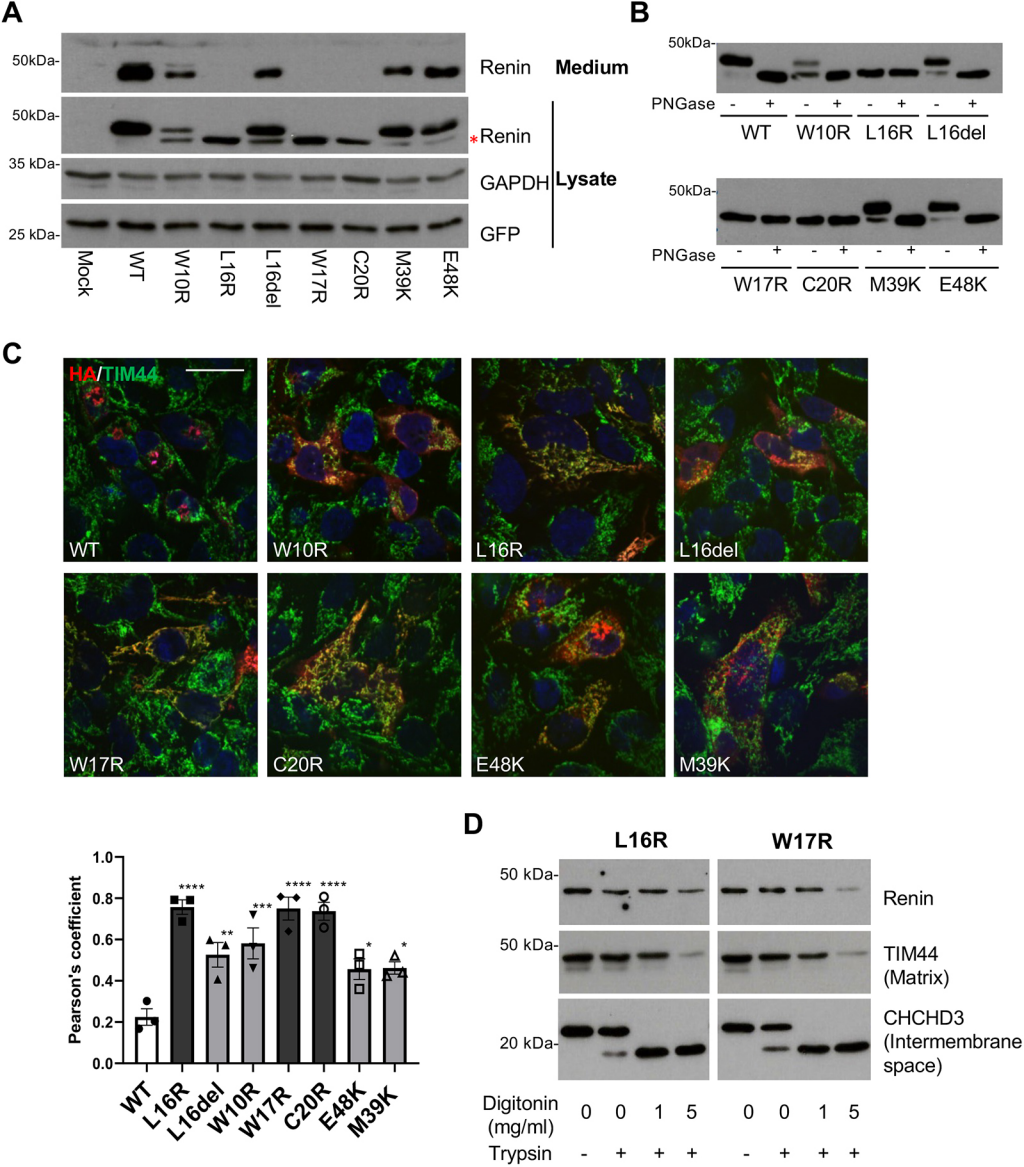
**Mitochondrial targeting of mutant renin isoforms.** (A) Western blot analysis showing renin expression in the lysates and conditioned media of transiently transfected Calu-6 cells. GAPDH is shown as a loading control. The presence of a non-glycosylated renin isoform (red asterisk) can be seen for all mutants. (B) Cell lysates were deglycosylated with PNGase F. The lower band indicated in panel A with the asterisk is not sensitive to PNGase F treatment, indicating the absence of N-glycans. (C) Immunofluorescence analysis in Calu-6 cells showing merged pictures of renin (red) and TIM44 (marker of mitochondria, green) with nuclei (DAPI, blue). Scale bar: 20 μm. Colocalisation of renin and mitochondria can be assessed by the presence of merged yellow signal. The graph below shows Pearson's correlation coefficient between signals of TIM44 and renin. *P<0.05; **P<0.01; ***P<0.001; ****P<0.0001; two-way ANOVA with Bonferroni post hoc test versus WT. Data are shown as mean±s.e.m. (*n*=3 independent experiments). (D) Protease protection assay performed on isolated mitochondria from HEK293 cells expressing p.L16R or p.W17R mutant isoforms. Western blot experiments showed digestion of renin isoforms by trypsin only when the inner membrane was permeabilised. This result suggests that mutant isoforms are imported within the mitochondrial matrix. Images are representative of three independent experiments.

To verify the results of prediction analysis, we transiently expressed renin isoforms with mutations located in the leader peptide (p.W10R, p.L16del, p.L16R, p.W17R and p.C20R) and in the pro-segment (p.M39K and p.E348K) in Calu-6 cells, a human carcinoma-derived cell line expressing human renin endogenously at low levels (Lang et al., 1996). Western blot analysis showed that the mutants p.L16R, p.W17R and p.C20R were not or were barely secreted, whereas all other mutant isoforms were found in the extracellular medium (Fig. 2A). Moreover, in cell lysates of all mutants, we detected a low-molecular-mass additional band (Fig. 2A) that corresponds to a non-glycosylated form of renin, as shown by resistance to PNGase F (an enzyme removing N-linked oligosaccharides from glycoproteins) digestion (Fig. 2B). We hypothesised that this band could correspond to mutant renin that fails to interact with the ER translocon and is synthesised in the cytosol. Consistent with *in silico* prediction, analysis of renin subcellular localisation by immunofluorescence indicated mitochondrial localisation of all mutant isoforms, as shown by colocalisation with the mitochondrial marker TIM44 (encoded by *TIMM44*) (Fig. 2C). Whereas some mutants in the leader peptide were fully mistargeted to mitochondria (such as p.L16R, p.W17R and p.C20R), others were partially mislocalised (such as p.W10R and p.L16del). Mitochondrial localisation could also be detected for mutants in the pro-segment, even if reduced compared to that of mutants in the leader peptide. This is consistent with western blot results showing the non-glycosylated band only for mutants that were fully relocalised to mitochondria, whereas an additional signal, corresponding to the glycosylated protein, was seen for mutants that were partly rerouted to mitochondria and partly co-translationally inserted in the ER to enter the secretory pathway and acquire N-glycosylation (Fig. 2A). Similar results were obtained in HEK293 cells, a cell line of kidney origin that was previously used to study the effect of the *REN* mutation p.L381P mapping to the mature part of the protein (Schaeffer et al., 2019; Živná et al., 2009, 2020) (Fig. S1).

As immunofluorescence experiments showed co-staining between mitochondria and mutant renin isoforms, we wondered whether this reflected actual import of renin inside the organelle or its association on the outer membrane. To address this question, we performed a protease protection assay on mitochondria isolated from cells stably expressing representative mutants in the leader peptide (p.L16R and p.W17R). Digestion of the mitochondrial preparations with trypsin in different permeabilisation conditions showed that mutant renin isoforms were susceptible to protease digestion only in conditions permeabilizing the mitochondrial inner membrane. Mutant renin was in fact digested in the same conditions as TIM44, a subunit of the inner translocase facing the matrix; although it was still protected in conditions allowing digestion of CHCHD3, a protein of the intermembrane space (Fig. 2D). These results demonstrate that mutant renin isoforms are rerouted to mitochondria and imported in the mitochondrial matrix.

### Identification of mutant preprorenin domains that are necessary for targeting to mitochondria

**Fig. 3.**
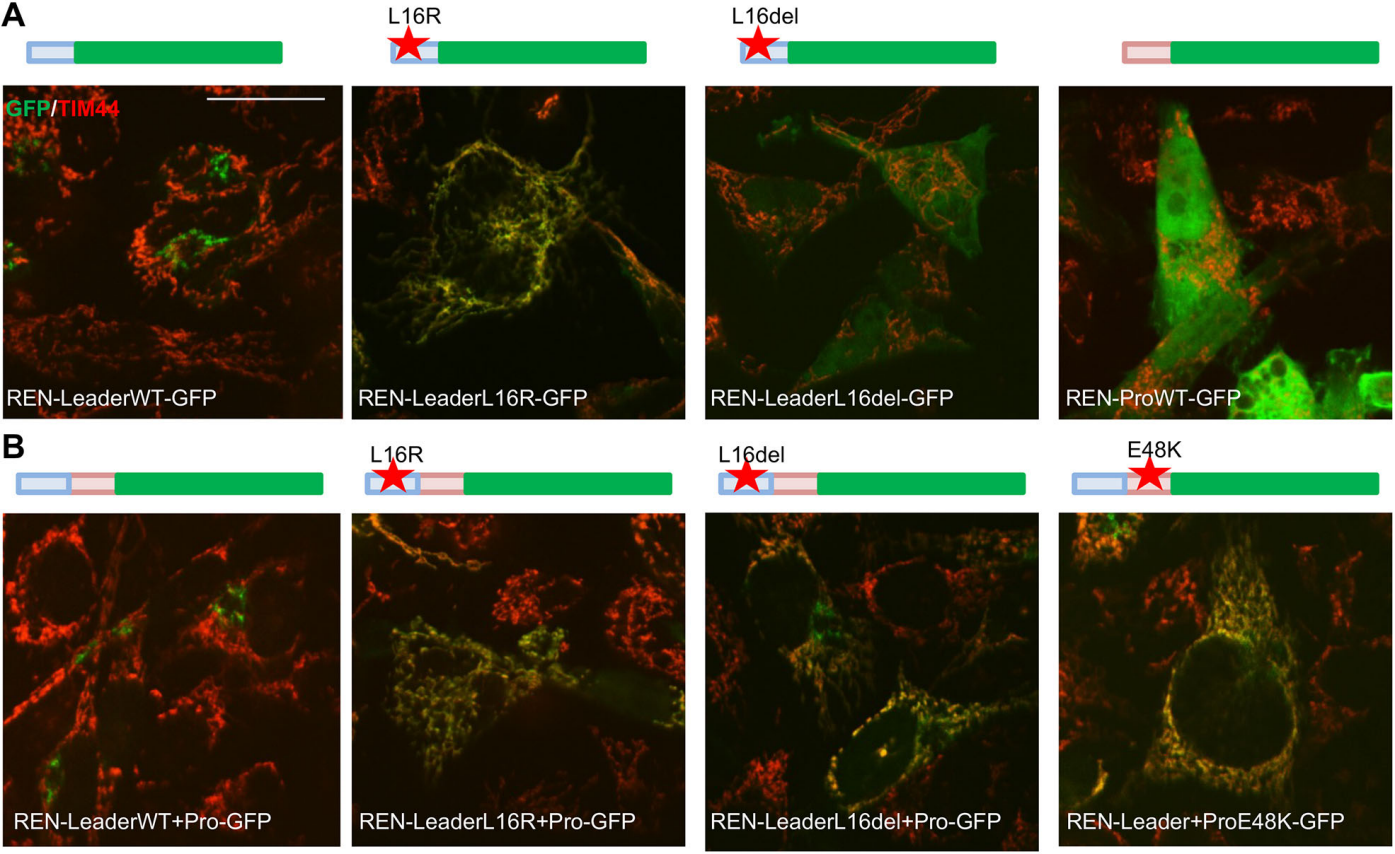
**Identification of the domain necessary for mistargeting to mitochondria of mutated renin isoforms.** Immunofluorescence analysis in Calu-6 cells showing merged pictures of the indicated GFP fusion constructs (green) and TIM44 (marker of mitochondria, red). Scale bar: 20 μm. A schematic representation of each construct is indicated above the picture. Leader peptide is depicted in blue, the pro-segment in pink and GFP in green. The red star indicates the insertion of an ADTKD mutation. Images are representative of three independent experiments.

We then wondered which renin domains are necessary for mutant renin import in the mitochondria. We addressed this question by employing chimeric constructs consisting of green fluorescent protein (GFP) fused to the renin leader peptide alone or to the leader peptide and pro-segment with WT or mutated sequence. We focussed on two representative mutations in the leader peptide showing complete (p.L16R) or partial (p.L16del) mitochondrial relocalisation and on one mutation (p.E48K) in the pro-segment. Through immunofluorescence analysis of transiently transfected Calu-6 cells, we observed that the mutation p.L16R was sufficient to convert the renin leader peptide into a mitochondrial-targeting sequence (MTS). GFP fused to the p.L16R leader peptide was indeed fully targeted to mitochondria, as assessed by complete colocalisation with the mitochondrial marker TIM44. The L16del leader peptide alone did not have such an effect on GFP localisation (Fig. 3A), although it led to partial re-localisation of GFP to mitochondria when the WT pro-segment was added (Fig. 3B). Notably, the pro-segment by itself did not lead to mitochondrial localisation of a fusion construct with GFP (Fig. 3A). The pro-segment carrying the p.E48K mutation, when fused to the WT leader sequence and GFP, induced partial targeting of GFP to mitochondria (Fig. 3B). We conclude that some ADTKD-*REN* mutations in the renin leader peptide modify it to a functional MTS, whereas others require the presence of the WT pro-segment domain to create a signal for mitochondrial targeting. When the leader peptide is not affected, introduction of a mutation in the pro-segment leads to mitochondrial localisation. The mature part of the protein is not necessary for mitochondrial localisation. Similar results were obtained in HEK293 cells (Fig. S2) demonstrating that the cellular effect of renin mutations is not restricted to one cell type.

### Expression of renin mutants leads to defective mitochondrial import

**Fig. 4.**
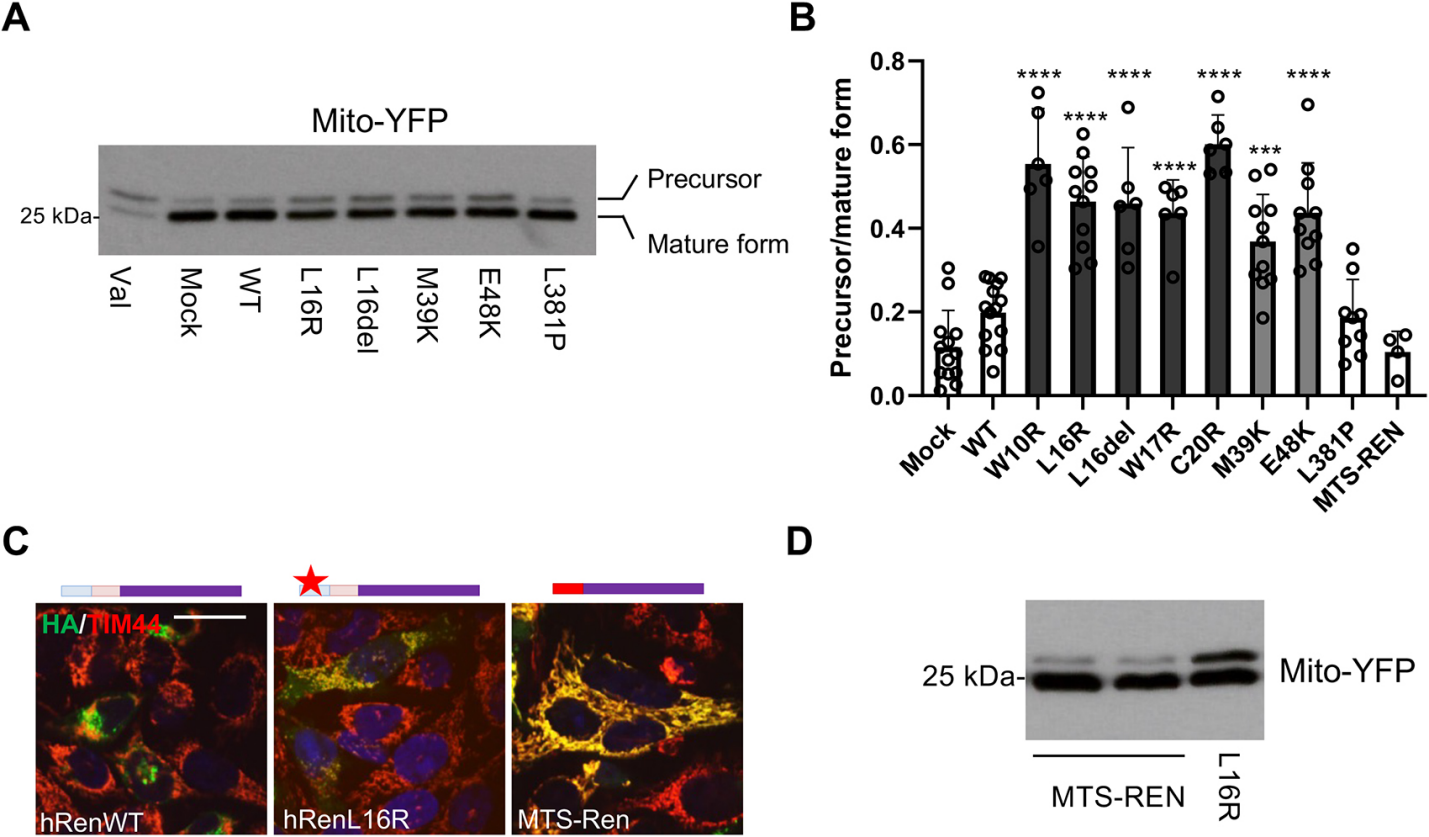
**Defective mitochondrial import upon expression of mutant renin isoforms.** (A) Representative western blot analysis showing Mito-YFP when co-expressed with the indicated renin isoforms. ‘Val’ indicates cells treated with valinomycin as a positive control of defective mitochondrial import. (B) Quantification of Mito-YFP import, measured as the ratio between precursor (YFP with the MTS, upper band) and mature (YFP without the MTS, lower band) forms. **P<0.01; ***P<0.001; ****P<0.0001; two-way ANOVA with Bonferroni post hoc test versus WT. Data are shown as mean±s.d. (*n*=4-14 independent experiments per group). (C) Immunofluorescence analysis showing merged images of the indicated renin construct (green), a mitochondrial marker (TIM44, in red) and nuclei (DAPI, blue). Yellow signals indicate the colocalisation of renin with mitochondria. Scale bar: 20 μm. A schematic of the transfected constructs is shown above each image. The leader peptide is depicted in blue, the pro-segment in pink, mature renin in purple and the MTS in red. The red star indicates the insertion of p.L16R mutation. MTS-REN is localised in the mitochondria as the p.L16R renin isoform. (D) Representative western blot analysis showing Mito-YFP when co-expressed with MTS-REN or the p.L16R renin isoform. Images are representative of at least three independent experiments.

We then analysed whether mutant renin isoforms interfere with the efficiency of the machinery of mitochondrial protein import, owing to the presence of a non-canonical MTS. To do so, we co-expressed mutant renin isoforms with yellow fluorescent protein (YFP) fused to a canonical MTS to efficiently route it to mitochondria (Mito-YFP). We quantified the rate of mitochondrial import of Mito-YFP by western blot analysis. Indeed, the MTS was cleaved once YFP entered the mitochondria, leading to a shift of molecular mass that allowed us to distinguish the precursor from the mature, imported form. Cells treated with valinomycin were used as a positive control for defective mitochondrial protein import. In these cells, accumulation of the precursor protein still containing the MTS could be clearly appreciated (Fig. 4A). Co-expression of Mito-YFP with renin isoforms mutated in the leader peptide or pro-segment led to an increased amount of the precursor form of Mito-YFP compared to that seen in mock cells or cells expressing WT renin (Fig. 4A,B). This result strongly suggests that the presence of mutant renin interferes with the import of mitochondrially localised proteins. The import defect was not observed when co-expressing the p.L381P renin isoform mutated in the mature part and previously shown to be retained in the ER (Schaeffer et al., 2019). We also analysed mitochondrial import of YFP when co-expressed with renin targeted to the mitochondria via a canonical MTS (MTS-REN). Although MTS-REN was indeed localised in mitochondria (Fig. 4C), its co-expression did not lead to an import defect of Mito-YFP (Fig. 4B,D). These results demonstrate that the expression of the investigated renin mutants leads to a mitochondrial import defect and that such an effect is due to the pre-pro-sequence of renin.

### Mitochondrial localisation of renin mutants leads to fragmentation of the mitochondrial network

**Fig. 5.**
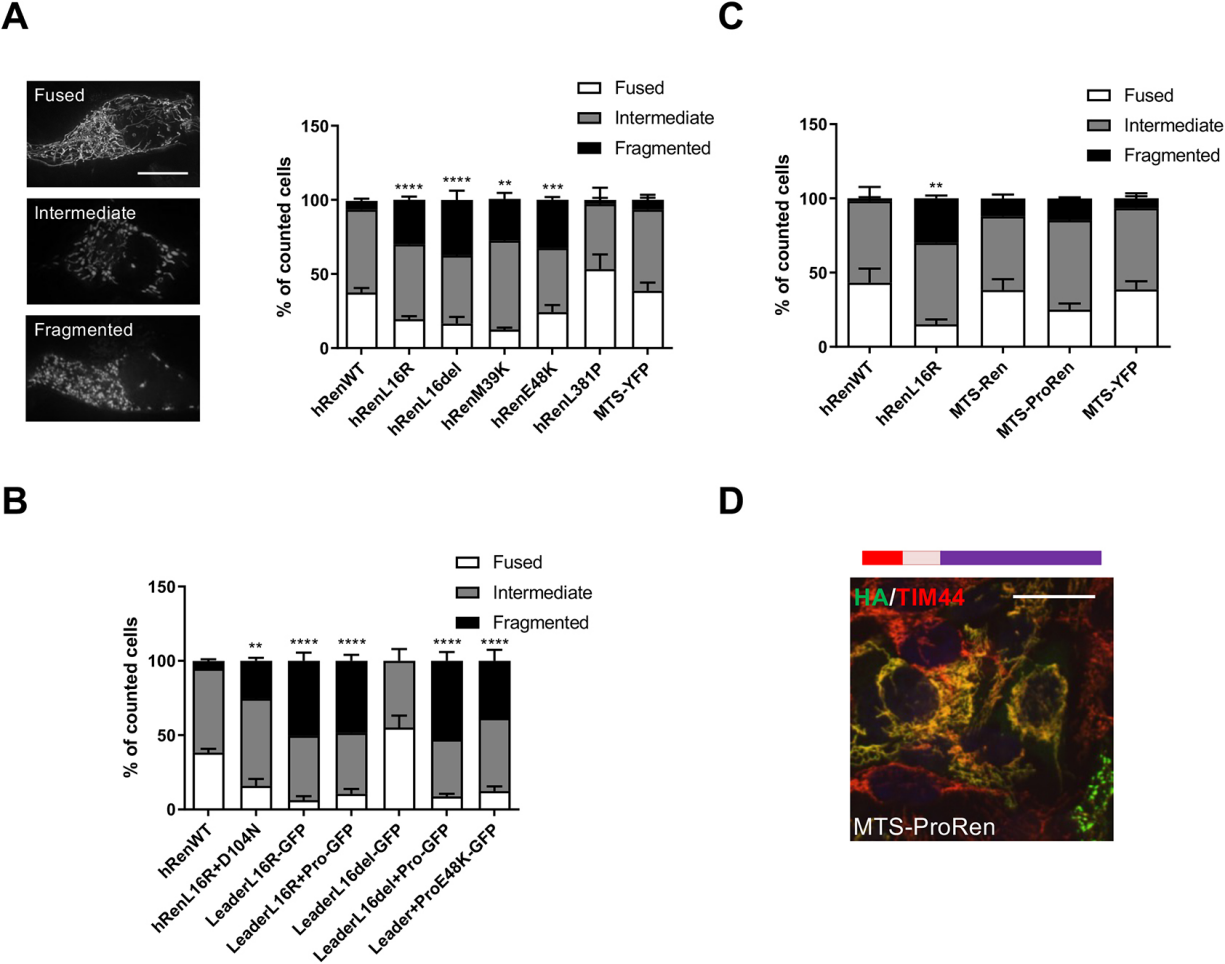
**Fragmentation of the mitochondrial network upon expression of renin mutants in the leader peptide or pro-segment.** (A) Analysis of the morphology of the mitochondrial network in Calu-6 cells expressing the indicated renin isoforms. Cells were divided in three classes based on the pattern of expression of YFP targeted to mitochondria when co-transfected with the indicated renin isoforms. Representative images of YFP in cells corresponding to each class are shown. Scale bar: 10 μm. **P<0.01; ***P<0.001; ****P<0.0001; two-way ANOVA with Bonferroni post hoc test (versus WT fragmented). Data are shown as mean±s.e.m. (*n*=3-15 independent experiments per group). (B) Morphology of the mitochondrial network was determined by analysing the pattern of expression of the YFP targeted to mitochondria when co-transfected with the indicated constructs. The mature part of renin is not necessary for mitochondrial fragmentation. **P<0.01; ****P<0.0001; two-way ANOVA with Bonferroni post hoc test (versus WT fragmented). Data are shown as mean±s.e.m. (*n*=3-9 independent experiments per group). (C) Morphology of the mitochondrial network was determined by analysing the pattern of expression of the YFP targeted to mitochondria when co-transfected with the indicated renin constructs. Mitochondrial targeting of renin was not sufficient to induce mitochondrial network fragmentation. **P<0.01; two-way ANOVA with Bonferroni post hoc test (versus WT fragmented). Data are shown as the mean±s.e.m. (*n*=3-4 independent experiments per group). (D) Immunofluorescence analysis showing the merged image of MTS-ProRen (green) with TIM44 (red) and nuclei (DAPI, blue). The yellow colour indicates colocalisation of renin constructs with mitochondria. A schematic of the construct is shown. The leader peptide is depicted in blue, the pro-segment in pink, mature renin in purple and the MTS in red. Scale bar: 20 μm. Images are representative of three independent experiments.

We next analysed the effect of mutant renin mistargeting to mitochondria on mitochondrial network morphology, as mitochondrial fragmentation has been reported upon induction of various stress signals (Eisner et al., 2018). All isoforms mutated in the leader peptide or the pro-segment, fully or partially localizing to the mitochondria, led to mitochondrial fragmentation. As a control, the mutant isoform p.L381P did not show a similar effect. Importantly, this effect was not observed when expressing an exogenous protein, YFP, fused to an MTS to efficiently route it to mitochondria (Mito-YFP) (Fig. 5A).

We then assessed the effect of chimeric constructs with the renin leader or leader and pro-segment sequences fused to GFP (Fig. 3). Notably, all constructs previously shown to be targeted to mitochondria induced fragmentation, whereas the fusion of GFP with the L16del leader peptide alone, which is not targeted to mitochondria, had no effect (Fig. 5B). These results show that the presence of a mutated region of renin that is sufficient to drive mitochondrial localisation – i.e. the mutated leader peptide with, for some mutations, the addition of the pro-segment – induces fragmentation, whereas the mature part of renin is not required. Consistently, fragmentation was not induced by mature renin targeted to the mitochondria via a canonical MTS (MTS-REN), even when the pro-segment was added, although these isoforms were properly localised to mitochondria (Fig. 5C,D).

### Interfering with ER translocation causes misrouting of WT renin to mitochondria

**Fig. 6.**
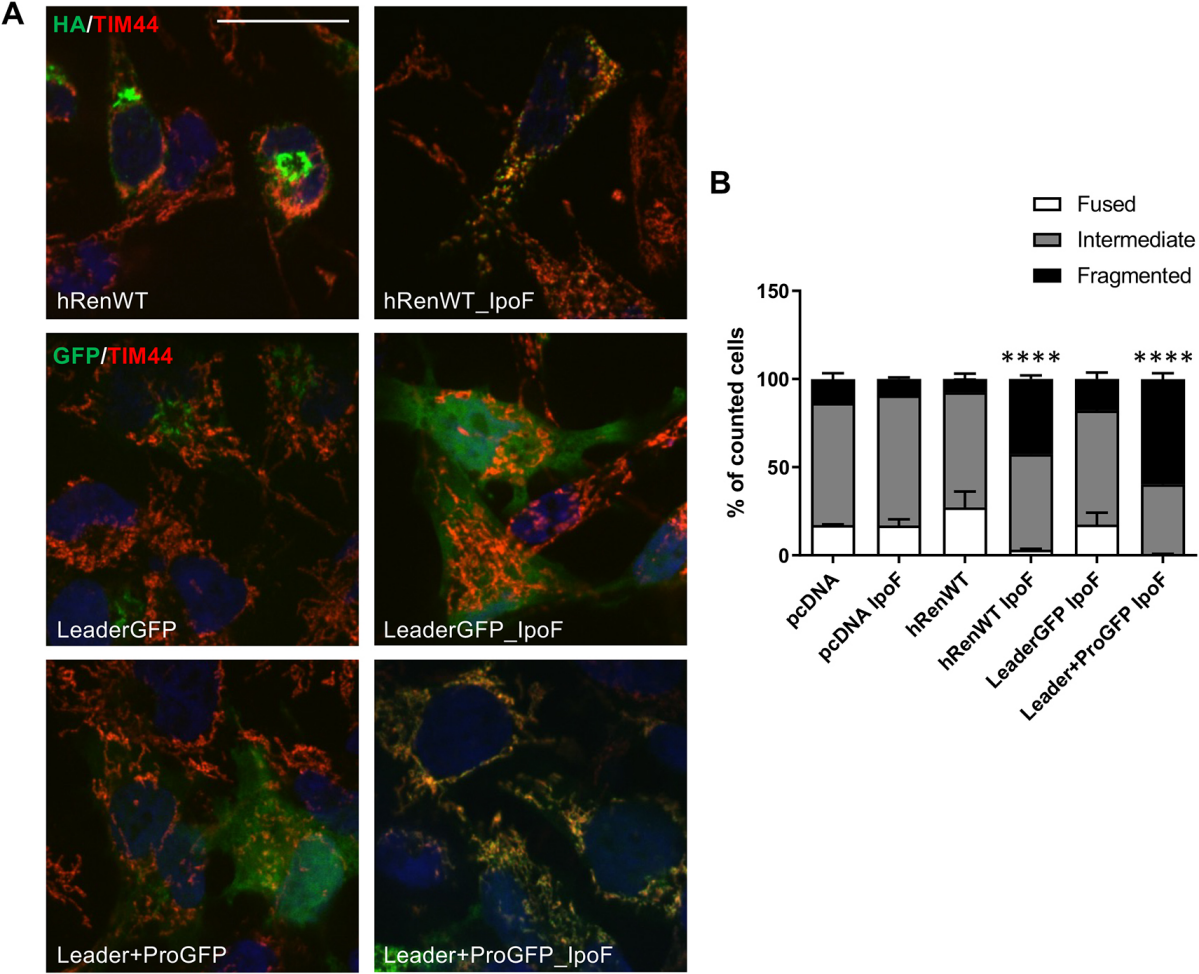
**Mitochondrial mistargeting of WT renin upon inhibition of ER translocation.** (A) Immunofluorescence showing merged images of the indicated renin constructs (green), a mitochondrial marker (TIM44, in red) and nuclei (DAPI, blue). Yellow colour indicates colocalisation of renin or GFP fused proteins with mitochondria. Scale bar: 20 μm. (B) Morphology of the mitochondrial network was determined by analysing the pattern of expression of YFP targeted to mitochondria when co-transfected with the indicated renin isoforms. ****P<0.0001; two-way ANOVA with Bonferroni post hoc test (versus WT fragmented). Data are shown as mean±s.e.m. (*n*=2-6 independent experiments per group).

To assess whether defective ER translocation could be the basis of mitochondrial misrouting observed for mutants of the leader peptide or pro-segment, we assessed the fate of WT renin when inhibiting ER translocation. To do so, we treated cells expressing WT renin with ipomoeassin F (IpoF), a chemical compound shown to interact with SEC61α, the pore-forming subunit of the SEC61 translocon, thus inhibiting ER translocation (Zong et al., 2019). Immunofluorescence analysis showed that after IpoF treatment, WT renin was misrouted to mitochondria (Fig. 6A). The use of GFP fused to the WT leader peptide or leader peptide and pro-segment demonstrated that the presence of the pro-segment was necessary for mitochondrial localisation upon inhibition of ER translocation. Indeed, GFP fused to the renin leader peptide alone accumulated in the cytosol after IpoF treatment (Fig. 6A).

Interestingly, mitochondrial localisation of WT renin or of preprorenin GFP fusion constructs led to mitochondrial fragmentation. This is consistent with findings on mutant renin isoforms, confirming that the presence of the leader peptide and pro-segment are sufficient to induce such an effect (Fig. 6B). These results also demonstrate that the WT pre-pro-sequence of renin has the same effect of the mutated sequences in conditions forcing their interaction with mitochondria.

### Leader peptide and pro-segment mutants do not affect trafficking of WT renin

**Fig. 7.**
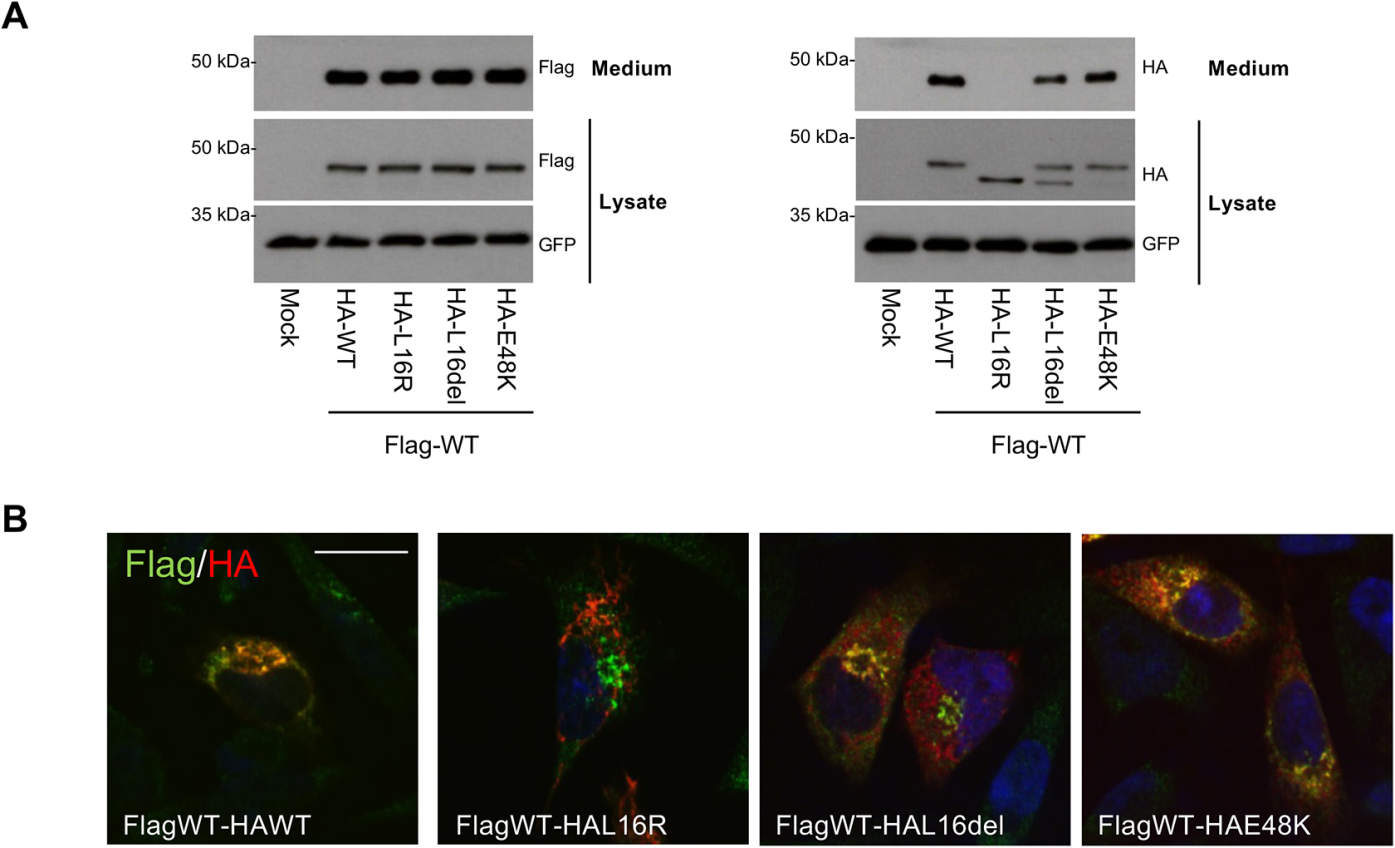
**Renin mutants do not have a dominant negative effect on trafficking and secretion of WT renin.** (A) Western blot analysis of co-expressed Flag- and HA-tagged renin isoforms in lysates and conditioned media of transfected CALU6 cells. GFP is shown as a control of transfection efficiency. (B) Immunofluorescence analysis showing the merged picture of Flag (green) and HA (red) renin isoforms in CALU6 cells. Wild-type renin cellular distribution is not affected by mutant renin co-expression. Scale bar: 15 µm. Images are representative of three independent experiments.

Finally, as ADTKD-*REN* patients are heterozygous for *REN* mutations and thus co-express both WT and mutant renin isoforms, we generated WT and mutant renin co-expressing cells to model the heterozygous condition. We used a dual-expression plasmid allowing co-expression of Flag-tagged WT renin and HA-tagged WT or mutant renin. Western blot analysis of lysates and conditioned medium showed the same signal pattern for the Flag-tagged WT protein when co-expressed with the HA-tagged WT isoform or different mutants (Fig. 7A). Consistently, immunofluorescence analysis showed that the localisation pattern of Flag-tagged WT renin is not different when co-expressed with HA-tagged WT or mutant isoforms (Fig. 7B). Hence, expression of mutant isoforms in the leader peptide or the pro-segment does not affect WT renin production and secretion, nor does it induce its accumulation as a non-glycosylated isoform.

## DISCUSSION

In this study, we identified a novel cellular phenotype for renin mutations associated with ADTKD-*REN* that are localised in the leader peptide and in the pro-segment domains. Expression of renin isoforms with such mutations indeed leads to full or partial mistargeting of mutant renin to mitochondria, an event that is associated with mitochondrial import defect and fragmentation of the mitochondrial network. These effects are specific for these types of mutation, as they are not observed for an ADTKD-*REN* mutation that maps in the mature part of the protein and that is retained in the ER. The renin pre-pro-sequence, carrying mutations either in the leader peptide or in the pro-segment, is necessary and sufficient to induce mitochondrial localisation and a subsequent mitochondrial phenotype (fragmentation and defective import). Additionally, the mature part of renin is dispensable and has no effect when delivered to mitochondria through a canonical MTS. Rather, the effect of renin mutants is reproduced in cells expressing WT renin, or just its pre-pro-sequence fused to GFP, when reducing the efficiency of ER translocation by inhibiting SEC61A1 activity. Taken together, our findings (1) reveal a common cellular phenotype for mutations mapping to the leader peptide or pro-sequence of renin that share common disease features; (2) suggest that localisation of the renin pre-pro-peptide to mitochondria, whether it is induced by an ADTKD-related mutation or by interfering with ER entry, leads to a mitochondrial phenotype; and (3) provide new insight into the molecular pathogenesis of the disease.

To date, only few studies reported the characterisation of localisation of renin mutants in the leader peptide and the pro-segment. The authors showed that leader peptide mutants fail to enter the secretory pathway and accumulate intracellularly (Bleyer et al., 2010; Živná et al., 2009, 2020), whereas pro-segment mutants accumulate in the ERGIC (Živná et al., 2020). Our results clarify the fate of leader peptide mutants, showing that they are partially or fully directed to mitochondria, hence uncovering a new cellular phenotype. The full (mutant p.L16R, p.C20R and p.W17R) or partial (mutant p.L16del) mitochondrial localisation is consistent with previous studies showing that these mutants do not enter or partially enter the secretory pathway, respectively (Živná et al., 2009, 2020). Along the same line, the partial mitochondrial localisation of mutants in the pro-segment does not exclude a possible accumulation in the ERGIC, as previously reported. In fact, in our study, mutants in the pro-segment can also be detected in the ERGIC as they are partly entering the secretory pathway (Fig. S3). Our findings demonstrate a common cellular phenotype (i.e. mistargeting to mitochondria, mitochondrial fragmentation and import defect) for renin mutants in the leader peptide and pro-segment. This is opposed to the effect of mutations in the mature protein, leading to ER retention of mutant proteins. These different cellular phenotypes have a clinical correlation (Živná et al., 2020), as mutations in the leader peptide and pro-segment are associated with early-onset disease, whereas mutations in the mature part lead to a milder adult-onset form of the disease (Schaeffer et al., 2019; Živná et al., 2020).

The association of different clinical features induced by mutations localised in different domains of the protein is reminiscent of mutations in the *INS* gene encoding insulin. Processing of insulin is very similar to renin, as it is synthesised as pre-proinsulin and activated in secretory granules to mature insulin, followed by release upon stimulation (Vasiljevic et al., 2020). Both dominant and recessive mutations in *INS* can lead to diabetes. In analogy to what was observed for renin, recessive *INS* mutations leading to early-onset diabetes are loss-of-function mutations. Instead, dominant mutations leading to early-onset diabetes, called mutant *INS*-gene-induced diabetes of youth (MIDY), or late-onset diabetes act via a toxic gain-of-function mechanism. Although heterogeneity of the clinical presentation partly depends on genetic and environmental factors, it is also explained by different effects of *INS* dominant mutations. Whereas mutations causing MIDY phenotype are characterised by proinsulin misfolding in the ER and exert a dominant-negative effect on the trafficking of the WT protein, mutations associated with late-onset diabetes, including mutations in the leader peptide, act via a different mechanism, leading to cytosolic accumulation downstream of defective ER targeting and translocation (Liu et al., 2015).

Recently, a mutation in the leader peptide of another protein of the renin-angiotensin system, angiotensin-converting enzyme (ACE), has been reported (Fila et al., 2020). Interestingly, similarly to *REN*, recessive loss-of-function mutations in *ACE* are associated with renal tubular dysgenesis. This new mutation associates with slowly progressive kidney failure and anaemia, which is not explained by the level of kidney failure, highly reminiscent of the phenotype reported for *REN* mutations in the leader peptide. However, characterisation of the subcellular localisation of this mutant was not investigated. Several studies reported that mutations in the leader peptide of different proteins interfere with ER translocation, thus leading to decreased secretion, for example, mutations in collagen X in Schmid metaphyseal chondrodysplasia (Chan et al., 2001) or cationic trypsinogen or serine protease inhibitor Kazal type 1 in autosomal dominant hereditary pancreatitis (Hegyi and Sahin-Tóth, 2017). Mistargeting to mitochondria of mutated proteins has already been reported in kidney diseases, although these are due to other types of mutations. For example, a mutation adding an MTS to a native peroxisomal-targeting sequence has been reported in enoyl-CoA hydratase and 3-hydroxyacyl CoA dehydrogenase (EHHADH), leading to renal Fanconi syndrome. Mislocalisation of EHHADH interferes with mitochondrial function by disturbing β-oxidation of long-chain fatty acids (Assmann et al., 2016). A peculiar mechanism of mitochondrial mistargeting has also been reported in primary hyperoxaluria, in which mutations in alanine:glyoxylate aminotransferase (AGT) expose a cryptic MTS. These mutations indeed inhibit AGT dimerization, which, in normal conditions, masks the MTS (Fargue et al., 2013).

Our study demonstrates that mutant renin isoforms in the leader peptide or pro-segment are imported in the mitochondrial matrix. We show that fusion with the mutated leader peptide of renin targets GFP to mitochondria, suggesting that a single point mutation in the leader peptide is sufficient to convert the ER-targeting sequence into a mitochondrial one. As for some mutations in the leader peptide (i.e. p.L16del), addition of the pro-segment is needed, and considering that mutations in the pro-segment also drive mitochondria mistargeting, it is likely that structural features within the nascent chain, besides its primary sequence, are also involved. Recent studies on insulin indeed showed that ER translocation does not only require an efficient leader peptide, but it also depends on downstream sequences (Yang et al., 2021). Moreover, structural elements within the nascent chains have been demonstrated to regulate alternative targeting of secretory proteins to mitochondria for diverse proteins, for example, the prion-like protein shadoo (Pfeiffer et al., 2013). We thus hypothesise that mistargeting to mitochondria of mutant renin is driven by decreased efficiency in ER targeting, which is associated with the presence of a downstream domain compatible with mitochondrial import. Mutations in the pre-pro-sequence could lead to a decreased interaction with the signal recognition particle (SRP). This could be the basis of mitochondrial mistargeting as it has been previously reported that absence of SRP leads to aberrant co-translational targeting to mitochondria of proteins normally targeted to the ER (Costa et al., 2018). This is consistent with our data showing that interfering with ER translocation by blocking SEC61A1 with IpoF is sufficient to induce targeting of WT renin to mitochondria. IpoF does not have a general impact on translocation, but rather a selective effect on a subset of secretory proteins, possibly because they have reduced ability to compete with IpoF for SEC61 binding (Roboti et al., 2021). In line with these findings, renin was recently shown to be among the proteins for which ER translocation is severely affected when SEC61A1 activity is genetically reduced by ADTKD-associated mutations (Sicking et al., 2022). Collectively, these results suggest that defective ER translocation and mitochondrial mistargeting of renin could be a mechanism shared by ADTKD-*SEC61A1* and ADTKD-*REN*. Moreover, these studies suggest that renin is among those secretory proteins that carry a weak leader peptide and hence are more likely to be affected in conditions affecting ER translocation.

**Fig. 8.**
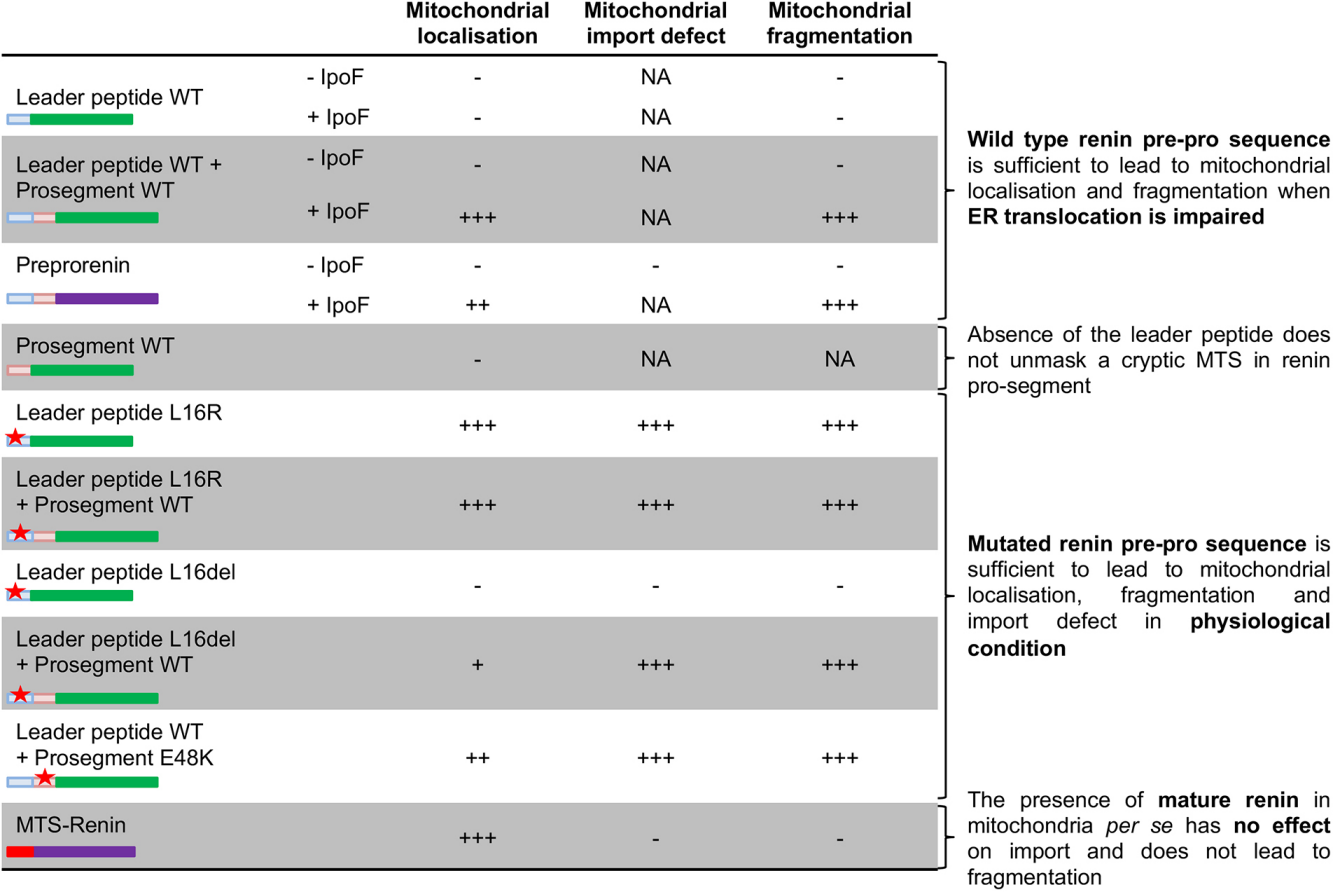
**Summary of the effects of preprorenin mutations depending on their position in the protein domains.** ‘+’ and ‘−’ indicate the presence and absence of the phenotype, respectively. NA, not assessed. A schematic representation of each construct is shown. The leader peptide is depicted in blue, the pro-segment in pink, a canonical mitochondrial targeting sequence (MTS) in red, the mature part of renin in purple and GFP in green. The red star indicates the insertion of an ADTKD mutation.

Our study highlights the importance of the renin pre-pro-sequence to induce a mitochondrial phenotype (Fig. 8). Mitochondrial import defect and fragmentation were indeed observed upon expression of GFP fused to the mutated leader peptide with, in some cases, addition of the pro-segment. Mitochondria fragmentation was also observed when forcing the interaction with mitochondria of WT renin or GFP fused to the WT renin pre-pro-sequence by inhibiting ER translocation (IpoF treatment). On the contrary, mitochondrial phenotypes were not observed by targeting mature renin to the mitochondrial matrix using a canonical MTS. This clearly demonstrates that the toxic effect is exerted by renin pre-pro-sequence interaction with mitochondria, whereas the mature protein and its activity are not required. Further studies are needed to understand the molecular bases of this toxic effect that could, for instance, be mediated by the interaction of renin mutants with components of the mitochondrial import machinery in the outer and/or inner membrane and the consequences of defective import on mitochondrial function. A similar mechanism has been described in Parkinson's disease for α-synuclein, which interacts with the import machinery to lead to defective import (Di Maio et al., 2016). This, in turn, leads to mitochondrial complex-I dysfunction, which is rescued by overexpression of translocases of the outer and inner membrane (Franco-Iborra et al., 2018).

Given the functional studies performed so far on mutations in the different ADTKD genes (i.e. *UMOD*, *MUC1*, *SEC61A1* and *REN*), ER stress and unfolded protein response have been proposed to play a central role in disease onset and progression (Devuyst et al., 2019; Mabillard et al., 2023; Schaeffer and Olinger, 2020). Our work, demonstrating mistargeting to mitochondria of renin mutants in the leader peptide and pro-segment, expands current knowledge of ADTKD pathogenesis. Of note, mitochondrial dysfunction, secondary to ER stress, has been reported in ADTKD-*UMOD* (Kemter et al., 2017). Additional studies are needed to characterise cell responses that are induced by mutants mistargeted to mitochondria and how this is eventually linked with the induction of inflammation and fibrosis in the kidney.

## MATERIALS AND METHODS

### Constructs

Human cDNA of a untagged renin clone (SC321832) was purchased from Origene (Rockville, MD, USA). Renin cDNA was subcloned in pcDNA3.1 (Thermo Fisher Scientific, Waltham, MA, USA) and HA was inserted at the C-terminal (Schaeffer et al., 2019). The indicated mutations were introduced in the pcDNA-hRenHA construct using the QuikChange Lightning Site-Directed Mutagenesis Kit (Agilent Technologies, Santa Clara, CA, USA). Primers were designed using the software QuikChange Primer Design program.

Fusion constructs between GFP and the leader or leader and pro-segment domains were obtained by PCR as follows. Two PCRs were performed to produce the sequences of GFP with the leader peptide, pro-segment or leader peptide and pro-segment. The two PCRs were designed in order to obtain two products with complementary ends, which could be used as template in a third PCR to obtain the final construct with the 5′ and 3′ ends at BamHI and XbaI sites, respectively, for cloning in the pcDNA3.1 vector.

MTS-YFP was expressed from the pYFP-Mito vector (Clontech, Mountain View, CA, USA; 6115-1). The mature part of renin (renin) or the mature part of renin fused to the pro-segment (prorenin) was amplified by PCR from the pcDNA-hRenHA vector and cloned under the MTS from subunit VIII of human cytochrome c oxidase in the pYFP-Mito vector in place of the YFP using the BamHI and NotI cloning sites.

All constructs were verified by sequencing before use and are available upon request. The primers used in this study are provided in Tables S1-S3.

### Cell culture conditions

HEK293 cells [American Type Culture Collection (ATCC); CRL-1573] were grown in Dulbecco's modified Eagle's medium (DMEM; Thermo Fisher Scientific) supplemented with 10% fetal bovine serum (FBS; Euroclone, Pero, Italy), 200 U/ml penicillin, 200 μg/ml streptomycin and 2 mM glutamine at 37°C, 5% CO_2_. Calu-6 cells (ATCC; HTB-56) were grown in Eagle's minimum essential medium (LGC Standards, London, UK; 30-2003) supplemented with 10% FBS, 200 U/ml penicillin and 200 μg/ml streptomycin at 37°C, 5% CO_2_. Cells were transiently transfected using Lipofectamine 2000 (Thermo Fisher Scientific) following the manufacturer's protocol. All cell lines were routinely tested by PCR for the absence of mycoplasma contamination. When indicated cells were treated with 100 nM valinomycin (Cayman Chemical, Ann Arbor, MI, USA; 10009152) for 20 h.

### Western blotting

Cells were incubated for 14 h in OptiMEM (Thermo Fisher Scientific) 36 h after transfection and lysed in Triton lysis buffer [50 mM Tris-HCl, pH 7.4, 150 mM NaCl, 0.5% Triton X-100, 10 mM NaF, 0.5 mM sodium orthovanadate, 1 mM glycerophosphate and protease inhibitor cocktail (Sigma-Aldrich)] for 1 h at 4°C under rotation, followed by centrifugation for 10 min at 17,000 ***g***. Soluble fractions were quantified by the Bio-Rad Protein Assay (Bio-Rad, Hercules, CA, USA). Conditioned medium was precipitated with four volumes of acetone and resuspended in PBS. When indicated, lysates were deglycosylated with PNGase F (New England Biolabs, Ipswich, MA, USA) according to the manufacturer's instructions. Then, 20 µg of each protein lysate and the equivalent of 200 µl of medium were loaded onto reducing SDS-polyacrylamide (10%) gels. Transblotted nitrocellulose membranes (Cytiva, Marlborough, MA, USA) were incubated with the indicated antibodies, followed by incubation with the appropriate horseradish peroxidase (HRP)-conjugated secondary antibody. Protein bands were visualised with the Immobilon Western Chemiluminescent Horseradish Peroxidase Substrate kit (Millipore, Billerica, MA, USA).

#### Antibodies

The following primary antibodies were used: mouse purified anti-HA.11 epitope tag antibody (1:1000; BioLegend, San Diego, CA, USA; 901502), mouse monoclonal anti-GAPDH antibody (1:1000; Santa Cruz Biotechnology, Dallas, TX, USA; sc-32233), rabbit polyclonal anti-GFP antibody (1:5000; Thermo Fisher Scientific; A11122) and rabbit polyclonal anti-CHCHD3 antibody (1:1000; Novus Biologicals, Littleton, CO, USA; NBP1-83656). The following secondary antibodies were used: Amersham ECL rabbit IgG, HRP-linked whole antibody (from donkey) (1:7500; Cytiva; NA934-1ML) and Amersham ECL mouse IgG, HRP-linked whole antibody (from sheep) (1:7500; Cytiva; NA931-1ML).

### Immunofluorescence

Cells grown on coverslips were fixed in 4% paraformaldehyde for 15 min, permeabilised for 10 min with 0.5% Triton X-100 and blocked for 30 min with 10% donkey serum. Cells were labelled for 1 h 30 min at room temperature with the indicated antibodies, followed by a 1 h incubation with the appropriate Alexa Fluor-conjugated secondary antibodies. Cells were stained with 4,6-diamidino-2-phenylindole (DAPI) and mounted using fluorescence mounting medium (DAKO, Agilent). All pictures were taken with an UltraVIEW ERS spinning disk confocal microscope (UltraVIEW ERS-Imaging Suite Software, Zeiss 63×/1.4; PerkinElmer Life and Analytical Sciences, Boston, MA, USA) All images were imported in Photoshop CS (Adobe Systems, Mountain View, CA, USA) and adjusted for brightness and contrast. Pearson's correlation coefficient between the signal of renin and that of TIM44 was calculated using the Fiji plugin Coloc 2 (Schindelin et al., 2012).

#### Antibodies

The following primary antibodies were used: mouse purified anti-HA.11 Epitope Tag antibody (1:500; BioLegend; 901502), rabbit recombinant anti-TIM44 antibody (1:500; Abcam, Cambridge, UK; ab194829) and rabbit affinity isolated anti-ERGIC-53/p58 antibody (1:500; Sigma-Aldrich; E1031). The following secondary antibodies were used: donkey anti-mouse IgG (H+L) highly cross-adsorbed secondary antibody, Alexa Fluor™ 488 (1:500; Thermo Fisher Scientific; A21202), donkey anti-mouse IgG (H+L) highly cross-adsorbed secondary antibody, Alexa Fluor™ 594 (1:500; Thermo Fisher Scientific; A21203), donkey anti-rabbit IgG (H+L) highly cross-adsorbed secondary antibody, Alexa Fluor™ 488 (1:500; Thermo Fisher Scientific; A21206) and donkey anti-rabbit IgG (H+L) highly cross-adsorbed secondary antibody, Alexa Fluor™ 594 (1:500; Thermo Fisher Scientific; A21207)

### Protease protection assay

Mitochondria were prepared as follows: ∼15×10^6^ cells were resuspended in 1 ml isolation buffer (225 mM mannitol, 75 mM sucrose, 1 mM EGTA and 5 mM HEPES) containing protease inhibitor cocktail (Sigma-Aldrich), diluted 1/1000 and lysed using a syringe. The lysate was centrifuged for 10 min at 600 ***g*** at 4°C. The supernatant was then centrifuged for 10 min at 7000 ***g*** at 4°C. The pellet was washed in 500 µl isolation buffer and centrifuged for 10 min at 7000 ***g*** at 4°C. The pellet containing the mitochondria was finally resuspended in 50 µl isolation buffer. Isolated mitochondria (50 µg) were permeabilised with the indicated amount of digitonin (BioVision, Waltham, MA, USA) to permeabilise only the outer or both the inner and outer mitochondrial membranes, and, where indicated, digested with 50 µg/ml trypsin for 30 min on ice. The reaction was stopped by addition of 10 mM phenylmethylsulfonyl fluoride.

### Mitochondrial network morphology

Calu-6 cells were plated on coverslips in 35 mm wells (500,000 cells/well) and transfected with 300 ng pYFP-Mito to express MTS-YFP and 1.2 µg of the plasmid expressing the indicated construct using 7 µl Lipofectamine 2000 (Thermo Fisher Scientific). After 24 h of transfection, the YFP signal was analysed by live imaging. Pictures were acquired with *z*-stacks of 0.2 µm using a DeltaVision microscope with a 100× objective and deconvolved. For each transfection, 20 fields were analysed, in which the number of cells with fused, intermediate or fragmented mitochondrial networks were counted.

For experiments performed in conditions of ER translocation inhibition, cells were treated for 14 h with 100 nM IpoF. IpoF treatment was started 30 h after transfection. IpoF was a generous gift from Prof. Wei Shi (Ball State University, IN, USA).

## Supplementary Material

10.1242/dmm.049963_sup1 Supplementary information
