# Supplementary material for: Leader peptide or pro-segment mutants of renin are misrouted to mitochondria in autosomal dominant tubulointerstitial kidney disease
Source: Dis Model Mech. 2023 Jun 7;16(6):dmm049963. doi: 10.1242/dmm.049963 (PMC10259838; doi:10.1242/dmm.049963)
Supplement: Supplementary information [file dmm-16-049963-s1.pdf]

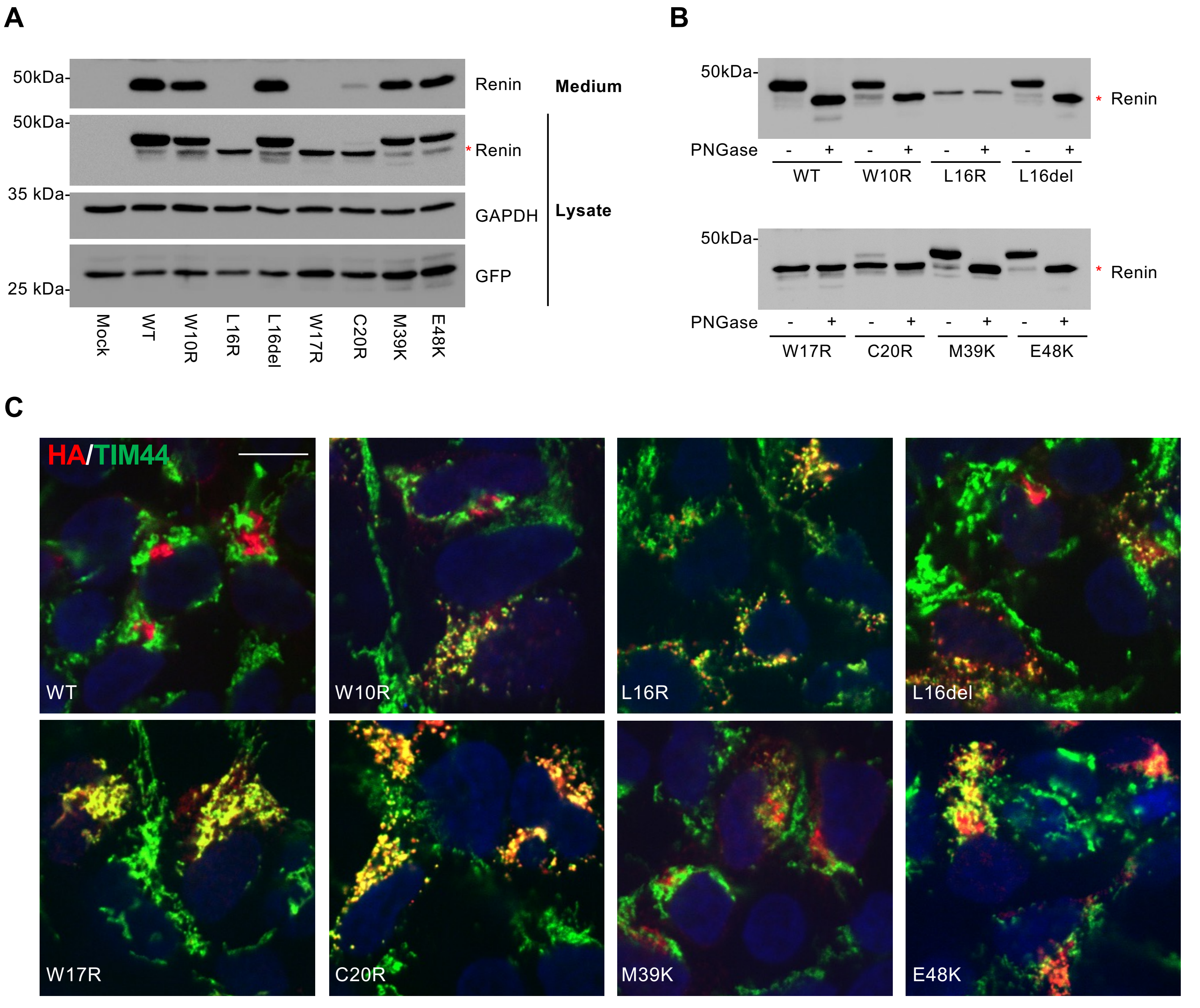

Fig. S1. Expression of the indicated renin isoforms in HEK293 cells. (A) Western blot analysis showing renin expression in HEK293 cell lysate and conditioned medium. GAPDH and GFP are shown as loading and transfection controls respectively. The presence of a non-glycosylated renin isoform (*) can be seen in all mutants. (B) Cell lysates were deglycosylated with PNGase F. The lower band indicated by * is not sensitive to PNGase F treatment demonstrating the absence of N-glycans. (C) Immunofluorescence analysis in HEK293 cells showing merged pictures of renin (red) and TIM44 (marker of mitochondria, green) with nuclei (dapi, blue). Scale bar: 10 µm. Co-localisation of renin and mitochondria can be assessed by the appearance of a yellow signal.

Disease Models & Mechanisms • Supplementary information

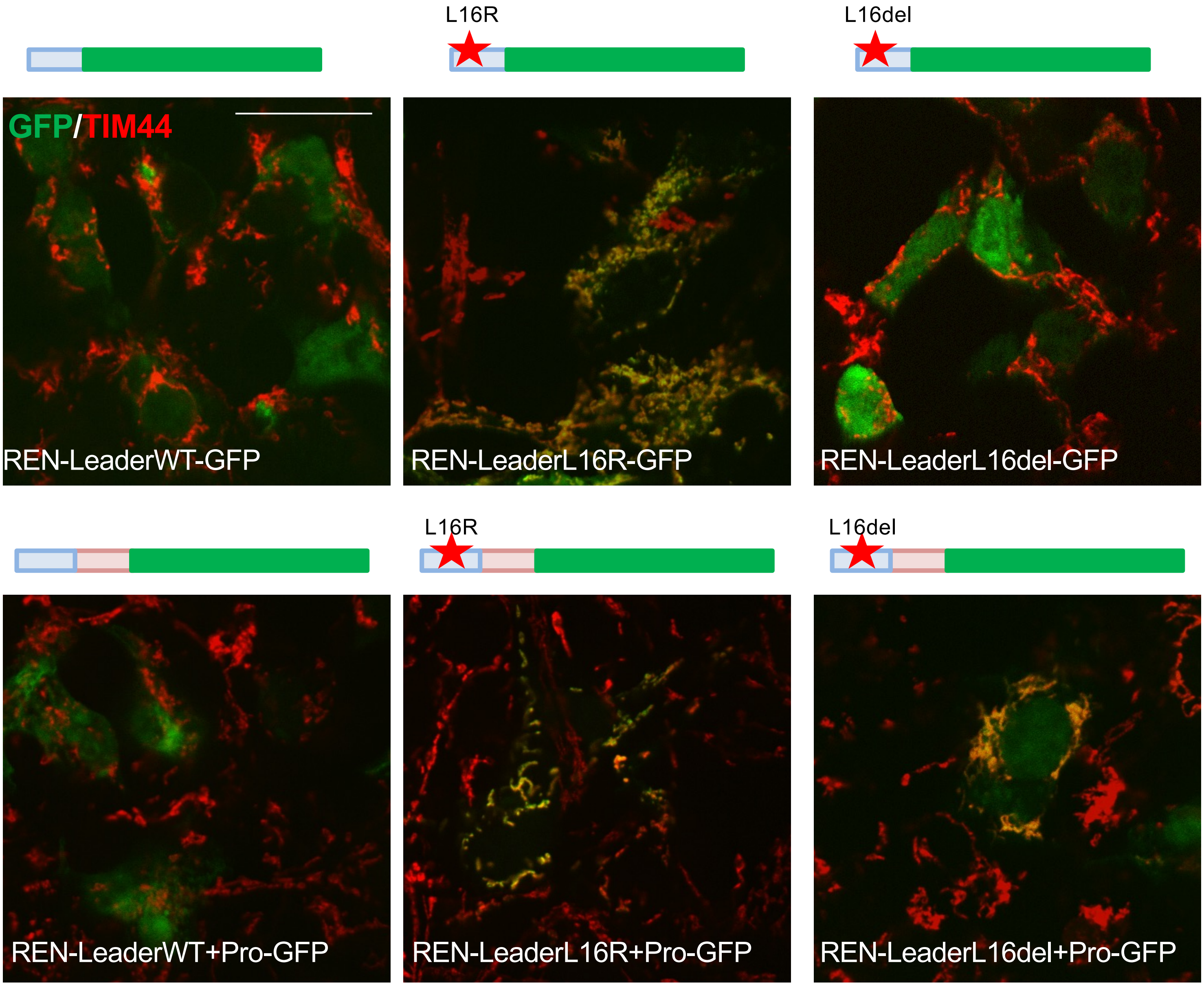

Fig. S2. Identification of the domain necessary for mistargeting to mitochondria of mutated renin isoforms in HEK293 cells. Immunofluorescence analysis showing merged pictures of the indicated GFP fusion constructs (green) and TIM44 (marker of mitochondria, red). Scale bar: 20 µm. A schematic representation of each construct is indicated above the picture. Leader peptide is depicted in blue, the pro-segment in pink and GFP in green. The red star indicates the insertion of an ADTKD mutation. Images are representative of 3 independent experiments.

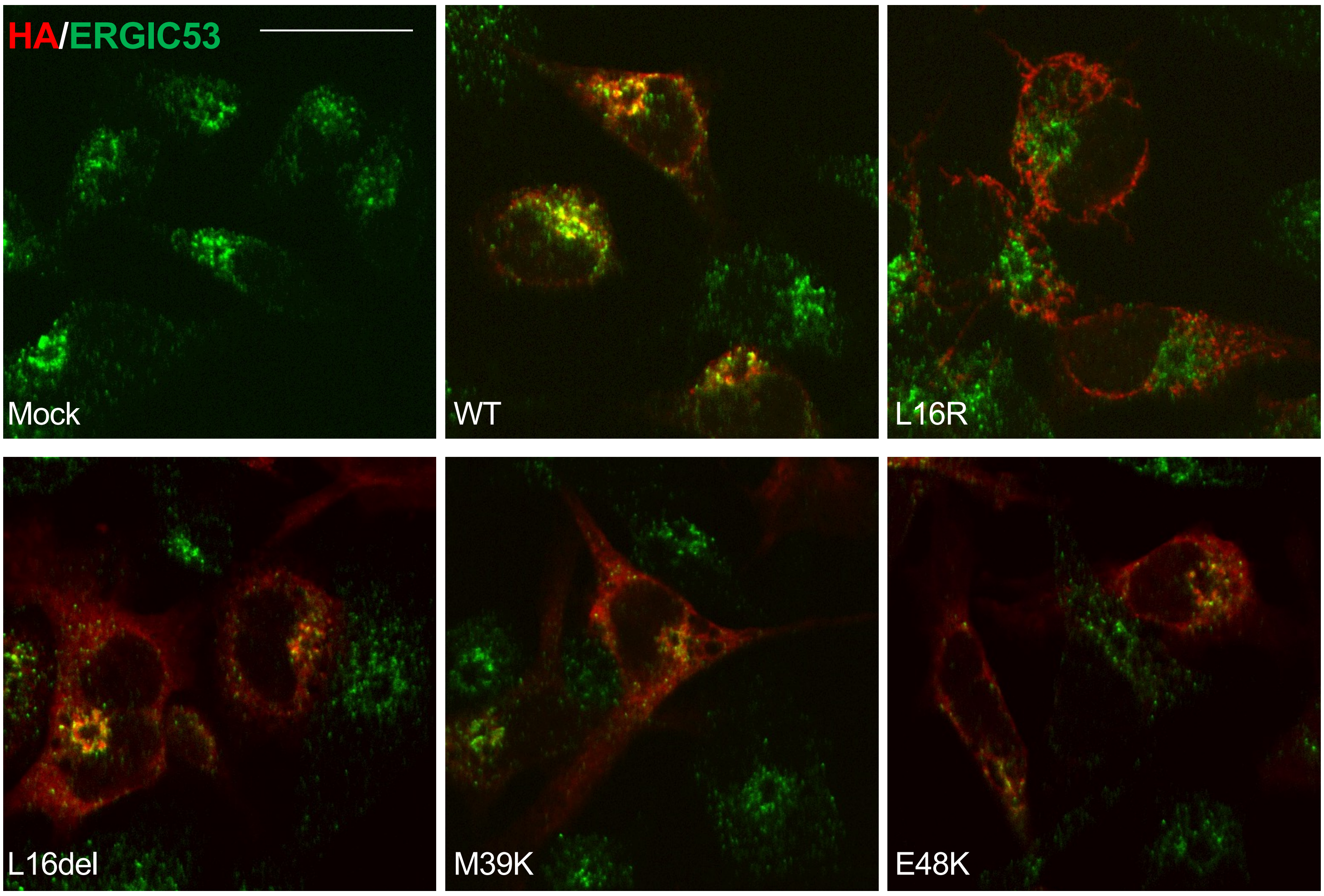

Fig. S3. ERGIC localization of the indicated renin isoforms in Calu-6 cells. Immunofluorescence analysis in Calu-6 cells showing merged pictures of renin (red) and ERGIC- 53 (marker of ERGIC, green). Scale bar : 20 µm. Co-localisation of renin and ERGIC can be assessed by the appearance of a yellow signal. No co-localisation was observed for mutant p.L16R that is fully misrouted to mitochondria. Images are representative of 3 independent experiments.

Disease Models & Mechanisms • Supplementary information

Table S1. Primers used for mutagenesis.

Mutation	Primers
hRen p.W10R	5′-AGGATGCCTCGCCGGGGACTGCTGC-3′
	5′ -GCAGCAGTCCCCGGCGAGGCATCCT-3′
hRen p.L16R	5′-TGCTGCTGCTGCGCTGGGGCTCCTG-3′
	5′-CAGGAGCCCCAGCGCAGCAGCAGCA-3′
hRen p.L16del	5′-GACTGCTGCTGCTCTGGGGCTCCTGT-3′
	5′-ACAGGAGCCCCAGAGCAGCAGCAGTC-3′
hRen p.W17R	5′- GCTGCTGCTGCTCCGGGGCTCCTGTAC-3′
	5′- GTACAGGAGCCCCGGAGCAGCAGCAGC-3′
hRen p.C20R	5′-TGCTCTGGGGCTCCCGTACCTTTGGTCTC-3′
	5′- GAGACCAAAGGTACGGGAGCCCCAGAGCA-3′
hRen p.M39K	5′- AACGGATCTTCCTCAAGAGAAAGCCCTCAATCCG-3′
	5′- CGGATTGAGGGCTTTCTCTTGAGGAAGATCCGTT-3′
hRen p.E48K	5′-CCGAGAAAGCCTGAAGAAACGAGGTGTGGACAT-3′
	5′-ATGTCCACACCTCGTTTCTTCAGGCTTTCTCGG-3′

Disease Models & Mechanisms • Supplementary information

Table S2. Primers used for generating the fusion constructs with GFP.

PCR	Primers
Leader peptide	5′-GCGGATCCGGGAAGCATGGATGGATGG-3′<br>5′-GCTCCTCGCCCTTGCTCACCGGGAGACCAAAGGTACAGG-3′
Leader peptide+Pro-segment	5′-GCGGATCCGGGAAGCATGGATGGATGG-3′<br>5′-GCTCCTCGCCCTTGCTCACCCTCTTCATGGGTTGGCTCCAC-3′
Pro-segment	5′-GCGGATCCGCTTATATGCTCCCGACAGACACCACC-3′<br>5′-GCTCCTCGCCCTTGCTCACCCTCTTCATGGGTTGGCTCCAC-3′
GFP for fusion with leader peptide only	5′-CCTGTACCTTTGGTCTCCCGGTGAGCAAGGGCGAGGAGC-3′<br>5′-GCTCTAGAGCTCACTTGTACAGCTCGTCCATGCCG-3′
GFP for fusion with pro-segment	5′-GTGGAGCCAACCCATGAAGAGGGTGAGCAAGGGCGAGGAGC-3′<br>5′-GCTCTAGAGCTCACTTGTACAGCTCGTCCATGCCG-3′
Fusion GFP-leader peptide	5′-GCGGATCCGGGAAGCATGGATGGATGG-3′<br>5′-GCTCTAGAGCTCACTTGTACAGCTCGTCCATGCCG-3′
Fusion GFP-leader peptide+pro-segment	5′-GCGGATCCGGGAAGCATGGATGGATGG-3′<br>5′-GCTCTAGAGCTCACTTGTACAGCTCGTCCATGCCG-3′
Fusion GFP-pro-segment	5′-GCGGATCCGCTTATATGCTCCCGACAGACACCACC-3′<br>5′-GCTCTAGAGCTCACTTGTACAGCTCGTCCATGCCG-3′

Table S3. Primers used for the generation of renin constructs fused with MTS.

PCR	Primers
Mature renin BamHI/NotI	5′-GCGGATCCACTGACACTTGGCAACACCACC-3′<br>5′-ATAAGAATGCGGCCGCCCCTCTAGAGCAGAGGGCC-3′
Prorenin BamHI/NotI	5′-GCGGATCCACTCCCGACAGACACCACC-3′<br>5′-ATAAGAATGCGGCCGCCCCTCTAGAGCAGAGGGCC-3′
